# Epigenetic Regulation of Hepatocellular Carcinoma Progression through the mTOR Signaling Pathway

**DOI:** 10.1155/2021/5596712

**Published:** 2021-05-25

**Authors:** Mengnan Guo, Ning Li, Jianxia Zheng, Wei Wang, Yan Wu, Xu Han, Jiapei Guo, Weixi Chen, Zekun Bai, Wen Bai, Jinghua Wu

**Affiliations:** ^1^Tangshan Maternal and Child Health Care Hospital, North China University of Science and Technology, Tangshan 063000, Hebei, China; ^2^North China University of Science and Technology Affiliated Hospital, Tangshan 063000, Hebei, China

## Abstract

Hepatocellular carcinoma (HCC), the most common type of primary liver cancer, is an aggressive tumor with a high mortality rate because of the limited systemic and locoregional treatment modalities. The development and progression of HCC depend on epigenetic changes that result in the activation or inhibition of some signaling pathways. The mTOR signaling pathway is essential for many pathophysiological processes and is considered a major regulator of cancer. Increasing evidence has shown that epigenetics plays a key role in HCC biology by regulating the mTOR signaling pathway. Therefore, epigenetic regulation through the mTOR signaling pathway to diagnose and treat HCC will become a very promising strategy.

## 1. Introduction

Hepatocellular carcinoma (HCC) is the fifth most common malignancy in men and the seventh most common malignancy in women worldwide and results in more than 700,000 deaths each year [1–3]. Further deterioration of HCC can be prompted by failure of early diagnosis, lack of effective therapeutic targets, and inadequate surveillance. Therefore, the development of novel diagnostic methods and the identification of novel targets for therapeutic intervention are urgently needed for the diagnosis and treatment of HCC [4].

Epigenetics is a heritable phenomenon that affects gene expression without altering the DNA sequence [5]. Epigenetics has been found to regulate gene expression mainly through four mechanisms: DNA methylation, histone modification, chromatin remodeling, and noncoding RNA regulation [6, 7]. Aberrant epigenetic regulation plays an important role in the development of human malignancies, including HCC. Therefore, better understanding of the abnormal epigenetic regulations in HCC may provide new options for the diagnosis and treatment of HCC. In addition, epigenetics also regulates signaling pathways such as the NF-*κ*B, RAF/MEK/ERK, JAK-STAT, Wnt, Notch, and MAPK as well as mTOR pathways [8–10]. Moreover, alterations in these signaling pathways can lead to changes in the biological traits of HCC cells to some extent [11]. Among these signaling pathways, the mTOR signaling pathway plays an important role in affecting HCC progression.

mTOR is involved in multiple signaling pathways that regulate cell proliferation, autophagy, and apoptosis in vivo [12]. There are two main mTOR signaling pathways: the classical PI3K/Akt/mTOR signaling pathway and the LKB1/AMPK/mTOR signaling pathway. Mutation, activation, and silencing of mTOR upstream genes, thereby regulating the mTOR pathway, affect the development process of HCC [7]. In addition, mTOR can also regulate protein synthesis by phosphorylating downstream target proteins p70s6k kinase (e. g., 6K1 and 4EBP1), which then regulate mRNA translation [13]. Therefore, changes in upstream and downstream genes of mTOR leading to dysregulation of mTOR signaling pathway will affect the progression of HCC. More than 70% of cancers are known to result in hyperactivation of mTOR. mTOR inhibitors have thus been widely studied for their use in cancer treatment, and some of these inhibitors (e. g., rapamycin, everolimus, doxorubicin, and sorafenib) are used for the treatment of HCC. However, clinical studies have shown that mTOR inhibitors have some limitations, such as low bioavailability and toxicity, and some cancers eventually acquire drug resistance [14–16]. Based on the limited efficacy of mTOR inhibitors, other novel drugs targeting mTOR need to be identified. Several mTOR inhibitors are currently under investigation for the treatment of HCC, and although many preclinical and clinical trial studies have been conducted, these inhibitors have not been applied. Therefore, the discovery of novel regulators of mTOR may become a new therapeutic target for HCC.

**Figure 1:**
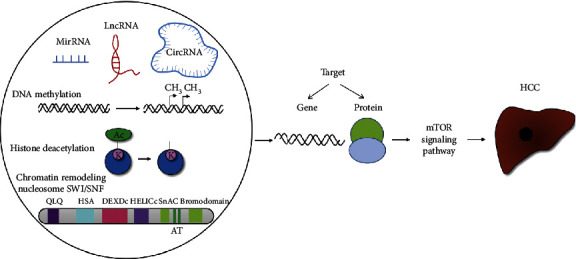
Epigenetic regulation of the mTOR signaling pathway in HCC.

In a review, we systematically summarize information regarding the regulation of the mTOR signaling pathway by factors mutated in epigenetic inheritance for the diagnosis and treatment of HCC (Figure 1).

## 2. ncRNAs

Noncoding RNA (ncRNA) refers to RNA that does not encode proteins [17]. Advances in sequencing technologies have led to the discovery of many ncRNA species, some of which are highly conserved, such as microRNAs (miRNAs), ultraconserved regions of transcription [18] and circRNAs (circRNAs), as well as ncRNAs that are not conserved between other species, such as long noncoding RNAs (lncRNAs) [19]. Studies have demonstrated that ncRNAs regulate cellular processes and pathways in developmental and pathological contexts [20]. In human diseases, particularly cancer, deregulated expression of ncRNAs can lead to changes in signaling pathways that can affect tumor development. Research has shown that ncRNAs are key players in human carcinogenesis and therefore may play potential roles in the diagnosis and treatment of cancer.

### 2.1. MiRNAs and HCC

MiRNAs are a diverse family of highly conserved ncRNAs with sizes that range from approximately 18 to 25 nucleotides. MiRNAs pair by complete or incomplete base complementation with the 3′ untranslated region of mRNA. This pairing leads to the degradation or inhibited translation of the target mRNA, with subsequent effects on protein expression. MiRNAs participate in the processes of cell growth, differentiation, development, proliferation, apoptosis, and metabolism [21, 22]. MiRNAs also play an important role in the physiological and pathological processes of cancer, including HCC. In recent years, a large number of studies have shown that the expression levels of miRNAs in HCC are upregulated or downregulated to varying degrees, suggesting that they play a role in various biological processes such as growth, proliferation, and apoptosis of HCC cells [23–26]. Therefore, miRNAs are very promising targets in the diagnosis and treatment of HCC.

#### 2.1.1. MiRNA Regulation of the PI3K/AKT/mTOR Signaling Pathway in HCC

HCC is the third leading cause of cancer-related death worldwide, and its incidence continues to rise. Although cirrhosis underlies most cases of HCC, many molecular pathways are closely associated with HCC carcinogenesis [27]. For example, activation or upregulation of the PI3K/AKT/mTOR signaling pathway can affect the occurrence and development of tumors [28]. The deregulation of this pathway has been shown to result from the deregulation of miRNAs. In HCC induced by mTOR signaling, the expressions of some miRNAs are decreased, and upregulation of these miRNAs is required to inhibit HCC development, while some miRNAs are increased in HCC (Table 1). A total of sixteen miRNAs (miR-1914 [29], miR-192-5p [30], miR-486-5p [31], miR-601 [32], miR-132 [33], miR-1207-5p [34], miRNA-133b [35], miR-144-3p [36], miR-26a [37], miR-21 [38], miR-199a-3p [39, 40], miR-758-3p [41], miR-494 [42], miR-125a [43], miR-345 [44], and miR-29a-3p [45]) have been identified as downregulated in HCC. Three miRNAs (miR-9-5p [46], miR-300 [47], and miR-181a [48]) were identified as upregulated in HCC.

#### 2.1.2. MiRNA Regulation of Other mTOR Signaling Pathways in HCC

In addition to miRNA-mediated regulation of the classical PI3K/AKT/mTOR signaling pathway, miRNAs can also affect HCC development through other mTOR signaling pathways.

In HCC, upregulation of miR-18a and miR-25 is associated with poor patient survival and promotion of HCC cell line proliferation. Sanchez-Mejias et al. [49] analyzed the predicted targets of some miRNAs and validated SOCS5 as a bonafide target of miR-18a and miR-25. Furthermore, the authors demonstrated that the SOCS5/miR-18a/miR-25 axis inhibits HCC development by regulating downstream mTOR signaling.

Zhou et al. [50] found that miR-100 downregulation was closely related to the development of HC. MiR-100 reduces the protein level of angiopoietin 2 (Angpt2) by targeting mTOR and blocking the mTOR-p70S6K signaling pathway, which in turn inhibits the formation of encapsulated tumor clusters (VETC), thereby eliminating VETC-dependent metastasis of HCC cells. These results indicate miR-100 as a new target for antimetastatic therapy of HCC.

Meanwhile, the experimental results of Dong et al. [51] showed that miR-223 could inhibit cell growth and promote apoptosis in HepG2 and Bel-7402 hepatoma cell lines and screened a novel miR-223 target, the Ras-related protein Rab-1 (Rab1). MiR-223 mediates mTOR signaling pathway inactivation by targeting Rab1, thereby inhibiting tumorigenesis and promoting HCC apoptosis. Therefore, miR-223 could be a potential therapeutic target for the treatment of HCC.

These studies indicate miRNAs can affect the progression of liver cancer by regulating the mTOR signaling pathway and provide a new approach for the diagnosis and treatment of liver cancer.

### 2.2. LncRNAs and HCC

Among the various types of ncRNAs, lncRNAs have received increasing attention. LncRNAs are defined as transcripts of more than 200 nucleotides that lack protein-encoding ability [52]. LncRNAs regulate the expression of genes by epigenetic regulation, transcriptional regulation, and posttranscriptional regulation in the form of RNA [53]. Therefore, lncRNAs have been shown to function as master regulators of gene expression and can play critical roles in various biological functions and disease processes in cancer, including HCC [54]. The genome-wide expression pattern of lncRNAs in HCC cells or tissues and their tissue-specific expression characteristics have been found by a number of studies, and lncRNAs hold promise as novel diagnostic biomarkers and therapeutic targets for HCC [55].

#### 2.2.1. LncRNAs Regulate the PI3K/Akt/mTOR Signaling Pathway in HCC

With the development of next-generation sequencing, more long noncoding RNAs (lncRNAs) were found. Initially, lncRNAs were considered as “noisy” transcripts or “dark matter” [56]. An increasing number of studies have revealed the indispensable role of lncRNAs in the dysregulation of signaling pathways in HCC [57]. The impact of lncRNAs on the progression of HCC through the PI3K/Akt/mTOR signaling pathway is relatively novel. Upregulation or downregulation of PI3K/AKT/mTOR-related oncogenic lncRNAs contributes to aberrant expression of transcriptional activators or oncoproteins, leading to aberrant regulation of the PI3K/AKT/mTOR pathway in HCC [58]. In addition, lncRNAs are aberrantly expressed in liver cancer and are significantly associated with metastasis, recurrence, prognosis, and chemoresistance of HCC [59]. Here, we summarize that 13 lncRNAs are dysregulated in HCC through the PI3K/Akt/mTOR signaling pathway. Twelve of the lncRNAs (lncRNA DCST1-AS1 [60], lncRNA OGFRP1 [61], lncRNA TMPO-AS1 [62], lncRNA MALAT1 [63, 64], lncRNA AK023948 [65], lncRNA HAGLROS [66], lncRNA CDKN2B-AS1 [67], lncRNA SNHG16 [68], lncRNA CASC11 [69], lncRNA DUXAP10 [70], lncRNA PICSAR [71], and lncRNA LINC00680 [72]) were upregulated in HCC, while lncRNA HULC [73] was downregulated in HCC (Table 2).

#### 2.2.2. LncRNAs Modulate the Role of Other mTOR Signaling Pathways in HCC

Several studies have shown that lncRNAs also regulate other mTOR signaling pathways in liver cancer. Ma and colleagues [74] found that abnormal expression of the lncRNA HEIH was associated with HCC cell growth and metastasis. HEIH was highly expressed in tumor tissue, and HEIH reduction significantly reduced Huh7 and Hep3B hepatoma cell viability, migration, and invasion and induced apoptosis. MiR-199a-3p was identified as a downstream effector of HEIH, and the functional effects of HEIH on Huh7 and Hep3B cells were attenuated when the miR-199a-3p expression was inhibited. In addition, reduced HEIH inhibited the activation of mTOR signaling by upregulating miR-199a-3p, indicating that HEIH may be a potential target for HCC.

Wei and colleagues [75] found significantly increased HOTAIR expression in 84 HCC tissues compared with nontumor tissues and determined the effect of HOTAIR on HCC cell-regulated glucose metabolism by examining lactate and glucose levels. The authors found that HOTAIR promotes glycolysis by upregulating glucose transporter isoform 1 (GLUT1) and activating mTOR signaling, while downregulation of HOTAIR inhibits this effect. This study revealed a new relationship between HOTAIR and glucose metabolism in HCC cells and indicated HOTAIR as a new target for the diagnosis and treatment of HCC.

Li et al. [76] found for the first time the upregulation of lncRNA-OR3A4 in HCC tissues and cell lines and identified OR3A4 as a promoter of HCC progression and angiogenesis. OR3A4 regulates HCC proliferation, metastasis, and angiogenesis through AGGF1/AKT/mTOR. The authors revealed that OR3A4 is a novel predicted target for HCC.

The lncRNA HIF1A-AS1 is overexpressed in HCC tissues and is associated with tumor size, TNM stage, and lymph node metastasis. Hong et al. [77] found that HIF1A-AS1 promotes hepatocarcinogenesis by activating autophagy through the HIF-1*α*/mTOR signaling pathway. The authors showed that HIF1A-AS1 is involved in regulating the progression of HCC and provided a potential direction for future HCC treatment strategies.

These reports indicate that dysregulation of lncRNAs can activate the mTOR signaling pathway to regulate the development of HCC, taking the understanding and potential use of lncRNAs in the diagnosis and treatment of liver cancer patients to a new level.

### 2.3. CircRNAs and HCC

CircRNAs are a novel class of ncRNAs characterized by a covalent closed-loop structure without a 5′ cap structure or a 3′ polyA tail [78]. Since the first discovery in viruses in the 1970s [79], circRNAs have begun to attract much attention, and research has been focused on their biogenesis, characteristics, functional mechanisms, and potential applications in clinical diagnosis and treatment. CircRNAs regulate gene expression at the transcriptional, posttranscriptional, and translational levels; they also regulate alternative splicing, sponge miRNAs, and sequester functional proteins. CircRNAs are involved in many pathological processes such as Alzheimer's disease, diabetes, atherosclerosis, and glioma [80–83]. CircRNAs play an important role in cancer growth, metastasis, recurrence, and treatment resistance [84].

The relationship between circRNAs and HCC has become a research hotspot in the past two years. CircRNAs were shown to have important regulatory roles in HCC development, and progression. In HCC, some circRNAs act as oncogenes and can promote the proliferation and migration of cancer, while other circRNAs act as tumor suppressors and can induce apoptosis of HCC cells. Although some studies have demonstrated that circRNAs can affect the progression of HCC, the roles of circRNAs on HCC remain largely unknown [85]. Therefore, the functions and mechanisms of circRNAs in HCC need to be further studied.

#### 2.3.1. CircRNAs Modulate the mTOR Signaling Pathway in HCC

CircRNA can affect the development and progression of HCC through the mTOR signaling pathway [86]. For example, Huang et al. [87] showed that circRNA-100338 activation of the mTOR signaling pathway through the circRNA-100338/miR-141-3p/RHEB axis is closely related to the poor prognosis of hepatitis B-related HCC. This study makes the connection between circRNA-100338 and mTOR signaling pathway in HCC cells and may provide a potential therapeutic target for HCC.

Sun et al. [88] found that three circRNAs—circRNA0004001, circRNA 0004123, and circRNA0075792—were upregulated in HCC blood samples using qRT-PCR. The expression of the three circRNAs was positively correlated with TNM stage and tumor size, and the three circRNA combination targeted a variety of miRNAs to participate in the mTOR signaling pathway, correlated with the development of liver cancer, and may be valuable diagnostic biomarker for HCC.

Zheng et al. [89] found that hsa-circ-0079929 was expressed at low levels in HCC. CircRNA-0079299 overexpression inhibited HCC growth and delayed cell cycle progression in vitro and in vivo but had no effect on cell migration and apoptosis. The inhibition of HCC growth by circRNA-0079299 is mediated by the PI3K/AKT/mTOR signaling pathway.

Although few reports have shown that circRNAs play a role in HCC through the mTOR signaling pathway, future studies clarifying their role in HCC development may lead to new approaches for the diagnosis and treatment of HCC.

## 3. DNA Methylation

DNA methylation is an important epigenetic modification that occurs mainly at the CpG islands of DNA and involves either hypermethylation or hypomethylation. Aberrant DNA methylation in cancer has been heralded as a promising target for the development of powerful diagnostic, prognostic, and predictive biomarkers [90]. For example, Abeni et al. [10] found that oncogene hypermethylation led to activation of mTOR signaling and inhibited tumor progression after sorafenib treatment. In addition, Liu et al. [91] found that the BCLB gene is methylated in HCC. Hypermethylated BCLB, which induces both apoptosis and autophagy in HCC cells through the AMPK-mTOR signaling cascade, plays a role in cancer suppression and has therapeutic implications for HCC patients.

### 3.1. Histone Modification

Histone modifications, as a class of epigenetic regulatory mechanisms that regulate gene expression, have received increasing attention because their modification pattern changes are closely related to the development of a variety of malignancies [92]. For example, cell differentiation and organismal development and abnormal modifications of histones contribute to diseases such as cancer [93]. Zhang et al. [94] found that acetazolamide (SIRT1), a NAD+-dependent histone deacetylase, exerts antioncogenic effects in HCC through the AMPK-mTOR pathway in the context of mutant p53. Wang et al. [95] found that inhibition of histone methyltransferase 3 (SMYD3) resulted in reduced AKT/mTOR signaling activity, which triggered deleterious effects on bladder cancer cells. In addition, Makarević et al. [96] found that decreased acetylation of H3 and H4 promotes prostate cancer cell development by activating the mTOR signaling pathway in prostate cancer. Sun et al. [97] found that inhibition of mTOR signaling enhanced trichostatin A and promoted histone acetylation in gastric cancer cell lines. To date, there have been no reports on the role of histone methylation and acetylation in liver cancer through the mTOR signaling pathway.

## 4. Chromatin Remodeling

The SWI/SNF complex, originally discovered in yeast 20 years ago, is a family of multi-subunit complexes that use the energy of ATP hydrolysis to remodel nucleosomes. Chromatin remodeling processes mediated by the SWI/SNF complex are essential for the regulation of gene expression in a variety of cellular processes, including differentiation and proliferation [98]. Many studies have found that the chromatin SWI/SNF complex plays an important role in malignant tumors. Zhou and his team [99] discovered SMARCD1, a subunit of the SWI/SNF complex, as a promising prognostic predictor that promotes liver cancer growth through the mTOR pathway.

## 5. Conclusions and Perspectives

Multiple studies have demonstrated that epigenetic changes play an important role in the development and progression of HCC. The mTOR pathway is involved in the growth and proliferation of HCC cells, and epigenetic regulation through mTOR signaling will affect HCC progression. As supported by increasing evidence, epigenetic regulators through the mTOR signaling pathway as ideal therapeutic targets for HCC are a potent future research direction. Although there have been major breakthrough in epigenetics in the treatment of HCC, some questions remain unanswered. For example, studies linking histone methylation and acetylation with the mTOR signaling pathway are scarce. Future studies should pursue this research direction to expand the exploration of strategies for HCC treatment targeting the mTOR pathway.

## Figures and Tables

**Table 1:** Dysregulated miRNAs in HCC.

	MiRNAs	Target	Expression	Reference
Sun et al.	MiR-1914	GPR39	Downregulated	[29]
Zhu et al.	MiR-192-5p	TRIP13	Downregulated	[30]
Youness et al.	MiR-486-5p	IGF-1R	Downregulated	[31]
Song et al., Liu et al.	MiR-601, miR-132	PIK3R3	Downregulated	[32, 33]
Zhao et al.	MiR-1207-5p	FASN	Downregulated	[34]
Wang et al.	MiR-133b	EGFR	Downregulated	[35]
Wu et al.	MiR-144-3p	SGK3	Downregulated	[36]
Sun et al.	MiR-26a	ST3GAL	Downregulated	[37]
Xia et al.	MiR-21	PTEN	Downregulated	[38]
Callegari et al., Lou G et al.	MiR-199a-3p	mTOR pathway	Downregulated	[39, 40]
Jiang et al.	MiR-758-3p	mTOR pathway	Downregulated	[41]
Pollutri et al.	MiR-494	mTOR pathway	Downregulated	[42]
Tang et al.	MiR-125a	mTOR pathway	Downregulated	[43]
Yu et al.	MiR-345	IRF1	Downregulated	[44]
Song et al.	MiR-29a-3p	Robo1	Downregulated	[45]
Dong et al.	MiR-9-5p	KLF4	Upregulated	[46]
Chang et al.	MiR-300	FOXO1	Upregulated	[47]
Chang et al.	MiR-181a	PTEN	Upregulated	[48]

**Table 2:** Dysregulated lncRNAs in HCC.

	LncRNA	Target	Expression	Reference
Li et al.	LncRNA DCST1-AS1	mTOR pathway	Upregulated	[60]
Chen et al.	LncRNA OGFRP1	mTOR pathway	Upregulated	[61]
Guo et al.	LncRNA TMPO-AS1	FOXK1	Upregulated	[62]
Peng et al.	LncRNA MALAT1	PI3k3′ noncoding region	Upregulated	[63]
Malakar et al.	LncRNA MALAT1	TCF7L2	Upregulated	[64]
Ye et al.	LncRNA AK023948	mTOR pathway	Upregulated	[65]
Wei et al.	LncRNA HAGLROS	MiR-5059/AGLROS axis	Upregulated	[66]
Zheng et al.	LncRNA CDKN2B-AS1	Let-7c-5p/NAP1L1 axis	Upregulated	[67]
Zhong et al.	LncRNA SNHG16	p62	Upregulated	[68]
Han et al.	LncRNA CASC11	PTEN	Upregulated	[69]
Sun et al.	LncRNA DUXAP10	GRP39	Upregulated	[70]
Liu et al.	LncRNA PICSAR	MiR-588	Upregulated	[71]
Shu et al.	LncRNA LINC00680	AKT3	Upregulated	[72]
Xin et al.	LncRNA HULC	PTEN	Downregulated	[73]

## Data Availability

The data used to support this study are included within this article.
